# Prevalence and correlates of foot pain in a population-based study: the North West Adelaide health study

**DOI:** 10.1186/1757-1146-1-2

**Published:** 2008-07-28

**Authors:** Catherine L Hill, Tiffany K Gill, Hylton B Menz, Anne W Taylor

**Affiliations:** 1 Rheumatology Unit, Queen Elizabeth Hospital, Woodville, South Australia, 5011, Australia; 2 Population Research and Outcome Studies Unit, Department of Health, Adelaide, South Australia, 5000, Australia; 3 Musculoskeletal Research Centre, Faculty of Health Sciences, La Trobe University, Bundoora, Victoria, 3086, Australia

## Abstract

**Background:**

Few population-based studies have examined the prevalence of foot pain in the general community. The aims of this study were therefore to determine the prevalence, correlates and impact of foot pain in a population-based sample of people aged 18 years and over living in the northwest region of Adelaide, South Australia.

**Methods:**

The North West Adelaide Health Study is a representative longitudinal cohort study of n = 4,060 people randomly selected and recruited by telephone interview. The second stage of data collection on this cohort was undertaken between mid 2004 and early 2006. In this phase, information regarding the prevalence of musculoskeletal conditions was included. Overall, n = 3,206 participants returned to the clinic during the second visit, and as part of the assessment were asked to report whether they had pain, aching or stiffness on most days in either of their feet. Data were also collected on body mass index (BMI); major medical conditions; other joint symptoms and health-related quality of life (the Medical Outcomes Study Short Form 36 [SF-36]).

**Results:**

Overall, 17.4% (95% confidence interval 16.2 – 18.8) of participants indicated that they had foot pain, aching or stiffness in either of their feet. Females, those aged 50 years and over, classified as obese and who reported knee, hip and back pain were all significantly more likely to report foot pain. Respondents with foot pain scored lower on all domains of the SF-36 after adjustment for age, sex and BMI.

**Conclusion:**

Foot pain affects nearly one in five of people in the community, is associated with increased age, female sex, obesity and pain in other body regions, and has a significant detrimental impact on health-related quality of life.

## Background

Foot pain has long been recognised as highly prevalent in older people, affecting approximately one in three people aged over 65 years [1-3]. In older people, foot pain is associated with decreased ability to undertake activities of daily living, problems with balance and gait, and an increased risk of falls [4-6]. The prevalence of foot pain in other age-groups, however, has not been as widely studied. The 1990 US National Health Interview Survey of 119,631 people aged over 18 years included a podiatry supplement and found that 24% of the sample reported foot "trouble" [7]. More recently, a random community-based sample of 3,417 people drawn from a general practice register in the UK found that 10% of study participants aged 18 to 80 years had "disabling" foot pain [8], and a community-based postal survey of 16,222 people aged over 55 years found that 18% reported joint pain, swelling and/or stiffness in their feet [9].

Although several studies in relatively small samples of older people have been undertaken [3,10,11], the prevalence of foot pain in the general Australian population has never been thoroughly examined. Therefore, the aims of our study were to determine the prevalence of foot pain in a population-based sample, to explore associations between age, sex, major medical conditions, other chronic joint symptoms and foot pain, and to assess the impact of foot pain on health-related quality of life.

## Methods

### Setting and study population

The North West Adelaide Health Study (NWAHS) was established in 2000 in the North-West region of Adelaide, South Australia [12]. The north-west region of Adelaide comprises approximately half of the population of the city of Adelaide and a third of the population of the state of South Australia. The regions also reflect the demographic profile of the state, covering a broad range of ages and socioeconomic areas. The study was designed in response to a need to assess the prevalence of priority conditions and examine their progression over time in a population-based community-dwelling cohort, to inform policy decisions about health care provision in South Australia.

Participants for Stage 1 of the study (which was conducted between 2000 and July 2003) were recruited randomly from the Electronic White Pages telephone listings and an initial telephone interview was conducted. Those within each household who were last to have a birthday and aged 18 years and over were interviewed and invited to attend a clinic assessment. The overall response rate for an interview and attending the clinic assessment was 49.4%.

Between 2004 and 2006, Stage 2 of the study was conducted. Where possible, all participants were contacted and invited to participate in a Computer Assisted Telephone interview (CATI), a self complete questionnaire and/or a clinic assessment. Stage 2 specifically focused on the collection of information relating to musculoskeletal conditions. Overall, n = 3,206 participants took part in the clinical assessment.

### Data collection

As part of the self completed questionnaire, information relating to demographics, self-reported prevalence of diabetes, levels of physical activity using the questions from the Australian National Health Survey [13] (the level of walking moderate or vigorous activity in the last two weeks) and health-related quality of life (the Medical Outcomes Study Short Form 36 (SF-36)[14] were collected. As part of the clinic assessment, height, weight, waist and hip circumference were measured, blood was taken, and as part of the CATI, the self reported prevalence of osteoporosis and cardiovascular disease were determined. Participants were also asked about the presence of knee, hip and back pain ("Have you had pain or aching in your knee/hip/back, either at rest or when moving, on most days for at least a month?").

**Figure 1:**
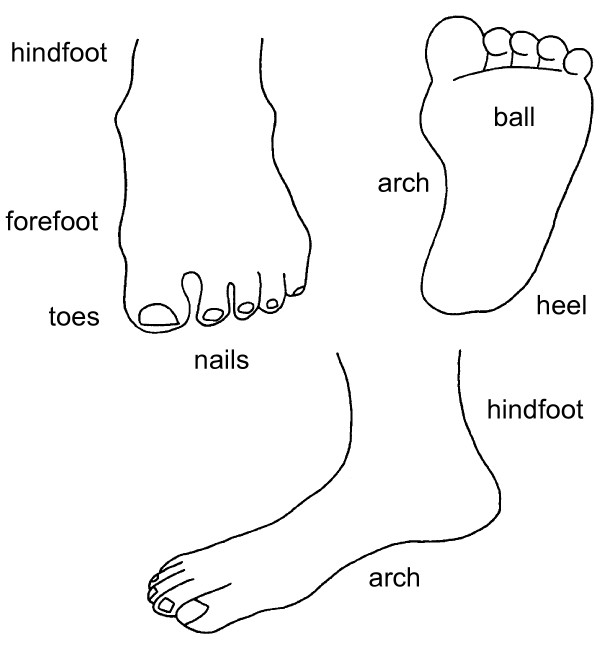
**Foot map used to document location of pain**.

All participants attending the clinic in Stage 2 were asked: "On most days do you have pain, aching or stiffness in either of your feet?" If they answered yes to this question, they were regarded to have foot pain. Participants who answered "yes" then indicated on a chart (Figure 1) the location of the pain. This was the same figure used in the Framingham Study [15].

### Statistical analysis

Data were weighted by age and sex, and probability of selection within the household, to the population of the north west suburbs of Adelaide. All analyses were undertaken using weighted data using SPSS Version 15. Frequencies were used to determine the prevalence of foot pain. Associations between foot pain, age and sex were undertaken using univariate logistic regression analyses to provide odds ratios. The association between foot pain and the remaining factors (BMI, selected chronic diseases and other areas of musculoskeletal pain) were determined using logistic regression analysis and including age and sex with in the model in order to adjust for these factors. As a result the impact of age and sex on the associations were determined. When examining the differences between males and females and age groups, in the proportion with foot pain at particular sites, Chi-square tests were undertaken. The mean health-related quality of life scores were determined using multiple analysis of variance (MANOVA). Age and sex were used as covariates in the analysis to adjust for their effects and the significant differences in scores between those with and without foot pain were determined. A significance level of *p *< 0.05 was used for all tests.

## Results

### Sample characteristics

Sample characteristics are shown in Table 1. The characteristics of the NWAHS cohort demonstrate that this is a relatively young, heavy cohort with 38% under 40 years and the mean BMI in the overweight range.

**Table 1:** Demographic characteristics of NWAHS, Stage 2 clinic assessment. Values are n (%) unless otherwise noted.

Variable	
Sex	
Male	1573 (49.1)
Female	1633 (50.9)
Age	
20 to 34 years	912 (28.4)
35 to 44 years	650 (20.3)
45 to 54 years	568 (17.7)
55 to 64 years	437 (13.6)
65 to 74 years	325 (10.1)
75 years and over	315 (9.8)
Body mass index (kg/m^2^) – mean (SD)	
Males	28.0 (5.0)
Females	27.7 (6.3)

### Prevalence and correlates of foot pain

#### Prevalence

Within the cohort, 558 (17.4%) of participants indicated that they had foot pain on most days over the past month. Of those with foot pain, 349 (62.5%) had bilateral foot pain and 209 (37.5%) had unilateral foot pain.

#### Foot pain, age, sex and weight

Associations between foot pain and age, sex and weight are shown in Table 2. Females were 40% more likely to report foot pain than males. Increasing age and a BMI classified as obese were factors associated with increased prevalence of foot pain. The presence of increased waist:hip ratio and absolute weight increase (per kg) was also associated with increased prevalence of foot pain.

**Table 2:** Associations of foot pain with sex, age and weight.

	n	%	OR (95% CI)	*p *value
Sex				
Male	237/1573	15.1	1.00	
Female	321/1633	19.6	1.38 (1.15–1.66)	0.001
Age				
20 to 34 years	93/912	10.2	1.00	
35 to 44 years	70/650	10.7	1.05 (0.76–1.46)	0.768
45 to 54 years	122/568	21.5	2.40 (1.79–3.22)	<0.001
55 to 64 years	105/437	24.0	2.78 (2.04–3.77)	<0.001
65 to 74 years	85/325	26.2	3.11 (2.24–4.32)	<0.001
75 years and over	83/315	26.4	3.14 (2.26–3.72)	<0.001
BMI (kg/m^2^)				
<30	326/2261	14.4	1.00	
>= 30	231/938	24.6	1.91 (1.57–2.31)*	<0.001
High waist:hip ratio†				
No	355/2434	14.6	1.00	
Yes	199/738	27.0	1.67 (1.36–2.06)*	<0.001
Weight (per kg)	-	-	1.021 (1.016–1.027)	<0.001

*adjusted for sex and age.

† men > 1.0, women >0.85.

#### Foot pain, chronic conditions, physical activity and pain in other regions

Associations between foot pain, chronic conditions, physical activity and pain in other body regions are shown in Table 3. There was an increased prevalence of foot pain amongst those with diabetes, cardiovascular disease and osteoporosis, however this did not reach significance following adjustment for age and sex. Of the participants with foot pain, 20.8% were sedentary compared to 16.4% of those without foot pain, however this difference was not statistically significant (odds ratio = 1.2; 95% confidence interval 0.9 – 1.4, *p *= 0.16). Foot pain was significantly associated with other joint pain, including knee, hip and back pain (all *p *< 0.001).

**Table 3:** Association of foot pain with chronic conditions and joint pain (adjusted for sex and age).

	n	%	OR (95% CI)	*p *value
Diabetes				
No	493/2949	16.7	1.0	
Yes	63/228	27.7	1.34 (0.98 – 1.85)	0.070
Cardiovascular disease				
No	482/2958	16.3	1.0	
Yes	53/204	25.8	1.10 (0.77–1.56)	0.611
Osteoporosis				
No	496/3047	16.3	1.0	
Yes	38/115	33.1	1.43 (0.94–2.18)	0.099
Physical activity				
Some level of activity	338/2058	16.4	1.00	
Sedentary	175/841	20.8	1.16 (0.94–1.43)	0.61
Knee pain				
No	383/2638	14.5	1.0	
Yes	146/500	29.1	2.40 (1.92–3.01)	<0.001
Hip pain				
No	437/2868	15.2	1.0	
Yes	95/285	33.4	2.36 (1.79–3.10)	<0.001
Back pain				
No	286/2220	12.9	1.0	
Yes	251/942	26.6	2.36 (1.94–2.86)	<0.001

NB: Don't know/not stated responses were excluded.

#### Foot pain location and sex

The location of foot pain according to sex is indicated in Table 4. Approximately equal numbers of males and females indicated pain in the forefoot, toes, ball, heel, hind foot and arch, with substantially less at the nails. Females were more likely to report pain in the toes and in the ball of the foot compared to males.

**Table 4:** Prevalence of foot pain by location and sex.

	Male		Female		Total	
	
	n	% (95% CI)	n	% (95% CI)	n	% (95% CI)
Hindfoot	67	28.1 (22.8–34.1)	82	25.5 (21.0–30.5)	148	26.6 (23.1–30.4)
Forefoot	73	30.6 (25.1–36.7)	109	34.0 (29.0–39.3)	182	32.5 (28.8–36.5)
Toes*	51	21.3 (16.6–26.9)	97	30.4 (25.6–35.6)	148	26.5 (23.0–30.3)
Nails	6	2.5 (1.2–5.4)	7	2.3 (1.1–4.6)	13	2.4 (1.4–4.0)
Arch	48	20.3 (15.7–25.9)	86	26.8 (22.2–31.9)	134	24.0 (22.2–31.9)
Ball*	43	18.2 (13.8–23.6)	93	28.9 (24.2–34.1)	136	24.3 (20.6–27.7)
Heel	42	17.6 (13.2–22.9)	75	23.3 (19.0–28.3)	116	20.9 (17.7–24.4)

* statistically significant difference between males and females (χ^2 ^test, *p *< 0.05)

#### Foot pain location and age

The location of foot pain according to age is shown in Table 5. Pain in the hindfoot region demonstrated a U-shaped relationship with age, with the highest prevalence noted in the 20–34 year and >75 year groups. Pain in the forefoot increased linearly until age 55–64, then levelled out across the remaining age-groups. Pain in the toes increased linearly with age. Pain in the arch was most prevalent in the 20–34 year age-group and decreased with age, with the exception of the >75 year group. Pain in the ball of the foot was similar across all age-groups, and pain in the heel decreased with age. There were insufficient cases to apply inferential statistics to pain in the nails.

**Table 5:** Location of foot pain, by age group (%).

	20 to 34 years	35 to 44 years	45 to 54 years	55 to 64 years	65 to 74 years	75 years and over
Hindfoot	37.4	24.4	21.0	22.5	23.6	32.8
Forefoot	11.0*	26.8	35.8	44.1*	39.1	35.4
Toes	6.2*	14.3*	27.8	28.9	36.4*	44.5*
Nails†	-	2.9	3.5	0.9	1.2	6.3
Arch	37.0*	31.6	26.4	15.5*	12.1*	22.6
Ball	28.5	25.1	26.1	23.1	22.4	20.2
Heel	30.5*	24.0	26.4	14.9	11.6*	16.6

* statistically significant difference compared to other age groups combined (χ^2 ^test, *p *< 0.05).

† statistical test not conducted due to small cell sizes

### SF-36 scores in participants with and without foot pain

Respondents with foot pain had significantly lower scores for all dimensions of the SF-36 compared to those without foot pain (Table 6).

**Table 6:** Mean (SD) SF-36 scores for those with and without foot pain.

	No foot pain (n = 2648)	Foot pain (n = 558)
Physical functioning*	82.1 (19.0)	71.3 (19.3)
Role physical*	80.5 (34.3)	64.4 (34.8)
Bodily pain*	76.0 (21.5)	60.8 (21.8)
General health*	70.7 (18.9)	61.2 (19.2)
Vitality*	64.8 (19.6)	55.1 (20.0)
Social functioning*	89.7 (19.8)	82.8 (20.0)
Role emotional*	90.4 (27.9)	82.2 (28.3)
Mental health*	78.9 (16.6)	71.6 (16.8)

*significant difference (*p *< 0.05), adjusted for age, sex and BMI.

## Discussion

Our study provides the first population-based estimates of foot pain in Australia. The findings indicate that approximately one in five people report foot pain, aching or stiffness, with a higher prevalence observed in females, those aged 50 years and over and those classified as obese. However, even in patients aged under the age of 45 years old, at least 10% reported foot pain.

The overall prevalence rate reported in this study is higher than that reported in the Cheshire Foot Pain and Disability Survey in the UK (10%) [8], but lower than that reported in the US National Health Interview Survey (24%) [7] and the Framingham Foot Study (28%) [15]. These differences can be attributed to variations in the definitions of foot pain used in each study. The Cheshire survey used the case definition of the Manchester Foot Pain and Disability Index (MFPDI), which requires participants to have current foot pain, pain lasting at least one month, as well as recording at least one disability item on the questionnaire [16]. As such, the MFPDI probably identifies more severe levels of foot pain than the question we used. In contrast, the US National Health Interview Survey recorded a wide range of foot conditions under the heading of foot "trouble" (including corns and calluses), some of which may not have been symptomatic [7]. The Framingham Foot Study required participants to have pain on "most days" [15].

The associations reported between foot pain and age, female sex and obesity are largely consistent with previous reports. Prevalence studies involving participants across a wide age range have consistently found that older people have much higher rates of foot problems [7,8,17], which has been attributed to the cumulative effects of ageing on the integumentary, vascular and musculoskeletal structures of the foot. Similarly, several studies have found that women have a higher prevalence of foot pain than men [8,11,18,19]. This has been attributed to the wearing of shoes with an elevated heel and narrow toe box, which has been shown to be associated with the development of corns, lesser toe deformities and hallux valgus (bunions) [20]. However, the higher prevalence of foot pain may also reflect sex differences in pain tolerance in general, as women are more likely to report musculoskeletal pain and pain interference at other body regions [21]. The association between foot pain and obesity can be partly explained by the significant increase in forces under the foot when walking in those who are obese [22] and the increased tendency for obese people to be flatfooted. Indeed, a recent case-control study indicated that those with chronic heel pain were three times more likely to be obese and four times more likely to have flat feet [23].

Although there was an increased prevalence of foot pain amongst those with self-reported diabetes, cardiovascular disease and osteoporosis, this did not reach significance following adjustment for age and sex. Previous studies have shown that foot problems are more common in older people with multiple chronic diseases [19,24,25], however in younger people foot pain is more likely to be related to overuse musculoskeletal conditions associated with physical activity (eg: plantar fasciitis) [7]. Indeed, although a strong linear relationship between foot pain and increased age was observed, this association was revealed to be considerably more complex when foot pain was stratified according to location. While pain in the toes and forefoot generally increased with age, pain in the arch and heel decreased with age, pain in the hindfoot region demonstrated a U-shaped relationship with age, and pain in the ball of the foot was similar across all age-groups. As no clinical assessments were undertaken to ascertain the underlying cause of the pain, the reasons for these variable patterns are uncertain. However, it could be speculated that foot pain in younger age-groups is more likely to be musculoskeletal in origin, whereas foot pain in older people is more likely to be caused by toe deformities, corns and calluses.

Irrespective of the underlying cause, our results indicate that foot pain has a significant impact on health-related quality of life. Participants who reported foot pain demonstrated lower scores on the SF-36, and this association persisted after adjusting for age, sex and BMI. Although significant associations between the presence of foot problems, self-reported disability [26] and inability to perform activities of daily living [4,18,19] have been reported in older people, the association reported between foot pain and reduced health-related quality of life across such a broad age range is a novel finding. Of particular interest, those with foot pain demonstrated lower scores for not only the physical and bodily pain components of the SF-36, but also the social functioning and mental health components. This finding suggests that the impact of foot pain extends beyond localised pain and encompasses much broader aspects of health-related quality of life.

The major strength of this study is the use of a population-based sample with excellent response rates over a broad age range. However, it is acknowledged that the study has several limitations. Firstly, we defined foot pain according to a single question rather than using foot-specific questionnaires, such as the Manchester Foot Pain and Disability Index [3,16] or Foot Health Status Questionnaire [27]. Secondly, we were unable to examine the participants' feet in the study. We asked participants to indicate on a diagram (see Figure 1) the location of the foot pain, but did not ask which specific types of foot problem each participant had, or undertake any measurements of foot deformity. However, studies conducted in older people have indicated that only a small proportion of clinically-determined foot problems are reported as symptomatic [15,28,29]. Stronger associations have been reported for foot pain and pain in other regions of the body [9,11,24,29,30], suggesting that foot pain may develop as part of a generalised form of osteoarthritis or systemic pain condition.

## Conclusion

This study indicates that although it has received relatively little attention in the epidemiological literature, foot pain is highly prevalent, even in young people, and has a significant detrimental impact on health-related quality of life. As the population ages and the prevalence of obesity increases, there is likely to be an increasing prevalence of foot pain. Further research is required to determine best practice models for managing foot pain and to determine whether the provision of foot care services, such as podiatry, are sufficient to meet this increasing demand.

## Competing interests

HBM is Editor-in-Chief of the *Journal of Foot and Ankle Research*. It is journal policy that editors are removed from the peer review and editorial decision making processes for papers they have co-authored.

## Authors' contributions

CLH, TKG and AWT conceived the study design, TKG conducted the statistical analysis, CLH and HBM interpreted the results and drafted the manuscript, and all authors read and approved the final manuscript.
